# Cartilage Tissue Engineering with Silk Fibroin Scaffolds Fabricated by Indirect Additive Manufacturing Technology

**DOI:** 10.3390/ma7032104

**Published:** 2014-03-13

**Authors:** Chih-Hao Chen, Jolene Mei-Jun Liu, Chee-Kai Chua, Siaw-Meng Chou, Victor Bong-Hang Shyu, Jyh-Ping Chen

**Affiliations:** 1 Department of Chemical and Materials Engineering, Chang Gung University, Kweishan, Taoyuan 333, Taiwan; E-Mail: chihhaochen5027@yahoo.com.tw; 2 Craniofacial Research Center, Department of Plastic and Reconstructive Surgery, Chang Gung Memorial Hospital, College of Medicine, Chang Gung University, Kweishan, Taoyuan 333, Taiwan; E-Mail: vbshyu@yahoo.com.tw; 3 NTU Additive Manufacturing Centre, School of Mechanical and Aerospace Engineering, Nanyang Technological University, Singapore 639798, Singapore; E-Mails: jolene-liu@imre.a-star.edu.sg (J.M.-J.L.); mckchua@ntu.edu.sg (C.-K.C.); msmchou@ntu.edu.sg (S.-M.C.); 4 Research Center for Industry of Human Ecology, Chang Gung University of Science and Technology, Kweishan, Taoyuan 333, Taiwan

**Keywords:** silk fibroin, indirect additive manufacturing technology, scaffold, chondrocytes, cartilage tissue engineering selective laser sintering

## Abstract

Advanced tissue engineering (TE) technology based on additive manufacturing (AM) can fabricate scaffolds with a three-dimensional (3D) environment suitable for cartilage regeneration. Specifically, AM technology may allow the incorporation of complex architectural features. The present study involves the fabrication of 3D TE scaffolds by an indirect AM approach using silk fibroin (SF). From scanning electron microscopic observations, the presence of micro-pores and interconnected channels within the scaffold could be verified, resulting in a TE scaffold with both micro- and macro-structural features. The intrinsic properties, such as the chemical structure and thermal characteristics of SF, were preserved after the indirect AM manufacturing process. *In vitro* cell culture within the SF scaffold using porcine articular chondrocytes showed a steady increase in cell numbers up to Day 14. The specific production (per cell basis) of the cartilage-specific extracellular matrix component (collagen Type II) was enhanced with culture time up to 12 weeks, indicating the re-differentiation of chondrocytes within the scaffold. Subcutaneous implantation of the scaffold-chondrocyte constructs in nude mice also confirmed the formation of ectopic cartilage by histological examination and immunostaining.

## 1. Introduction

Silk fibroin (SF) has received attention as a scaffold material for tissue engineering (TE), due to its excellent processability, high biocompatibility, controllable biodegradability and unique mechanical and thermal properties [1–3]. This protein-based biopolymer is composed of highly repetitive homogenized amino acid sequences and has been used in its native state or processed into films, foams or electrospun nanofibrous membranes. Native and regenerated SF fibers have been used as sutures or weaved as meshes for the replacement of ligaments and tendons, while SF films and foams have shown potential in treating ocular surface disorders, bone and cartilage ailments [4–6]. The possibility to modify the secondary structure of SF has also been acknowledged as a strategy to achieve desirable material properties. The conformation transition of SF with the formation of anti-parallel β-sheets allows improvement of cell adhesion, due to the change in surface roughness, the stiffness of the scaffold and the tailoring of degradability [7–9].

Production of SF scaffolds is most commonly based on traditional manufacturing methods, such as thermal induced phase separation, salt-leaching or gas foaming [10,11]. Since the size and interconnectivity of pores within SF scaffolds can have a direct impact on the behavior of seeded cells, while the geometry and architecture of the pore design is correlated with the mechanical properties of the scaffold, control over these factors has been an extensively investigated issue [12,13]. Solvent type, the inclusion of porogens, freezing temperature and concentration of SF are common parameters that are varied during processing in order to yield scaffolds with the desirable architectures and mechanical properties. For instance, SF scaffolds made using the combination of sucrose/hexafluoroisopropanol exhibited significantly weaker mechanical properties and slower degradability when compared to salt/water-derived constructs [14]. The effects of freezing temperature and silk concentration were also found to influence the porosity and pore size of SF scaffolds, which would eventually affect processes, such as cell migration and proliferation [15].

Although SF scaffolds manufactured using traditional methods have proven viability in TE applications, there is limited focus on the incorporation of complex and controllable architectures into SF scaffolds. To engineer tissue blocks with increasing size, issues concerning nutrient transport, waste removal and cell penetration within the deeper regions of the scaffold need to be resolved [16]. A proposed solution to this issue would be pre-designed channels to support mass transfer. Hence, the incorporation of complex and controlled architectures into SF scaffolds may augment TE development based on this biomaterial.

Additive manufacturing (AM) technology can be used to engineer tissue replacements that are structurally complex. In contrast to conventional methods, AM technology is capable of producing precise architectural features using a layer-by-layer approach based on computer-aided design (CAD) [17,18]. The ability to control architectural features also allows the design and customization of scaffolds to suit patient’s specific needs on the scale of gross morphology. Nonetheless, direct AM approaches involve harsh processing conditions, such as high temperature and the use of toxic solvents, and discourage the use of naturally-derived biomaterials [19].

The indirect AM approach integrates both traditional and advanced TE techniques, which provides both micro- and macro-structural features, respectively [20–26]. The procedures for the indirect AM approach include: (1) fabrication of a negative mold using an AM system; (2) casting of molten or aqueous biomaterial into the negative mold; and (3) removal of the sacrificial mold. The negative, sacrificial molds are commonly fabricated using inkjet printing technology or stereolithography with building materials that are soluble in non-toxic organic solvents [27].

The aim of this study is to evaluate the potential of an SF scaffold produced through an indirect AM method for cartilage TE. The overall shape and macro channels of the SF scaffolds in this work were completely under user control and manufactured using an indirect AM technique, which is rarely considered for protein-based scaffold biomaterials. The resulting three-dimensional (3D) construct was first evaluated for its chemical structure and thermal properties to ensure that the intrinsic properties of SF were maintained. The SF constructs were subsequently assessed for their potential to regenerate neo-cartilage tissues by *in vitro* and *in vivo* studies with porcine articular chondrocytes.

## 2. Results and Discussion

### 2.1. Scaffold Preparation and Characterization

**Figure 1.**
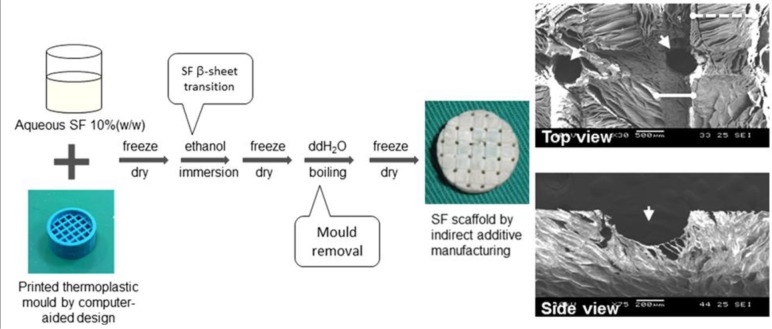
A printed thermoplastic mold through computer-aided design was first manufactured and a silk fibroin (SF) scaffold was obtained using the indirect additive manufacturing technique. The top view (bar = 500 μm) and side view (bar = 200 μm) of the circled area in the SF scaffold by scanning electron microscopy are shown. Arrowheads indicate penetrating channels. Solid and dotted lines demonstrate channel and inter-channel regions, respectively.

TE involves the development of temporary physical supports, or scaffolds, for tissue regeneration. The scaffolds are required to be made of biocompatible materials that can promote cell attachment and should possess adequate architectural components and features, such as optimal porosity, pore size and interconnectivity. This is to facilitate cell migration and mass transport of essential nutrients, while featuring optimal mechanical and degradability characteristics. In this study, SF scaffolds were produced through indirect AM technology, incorporating both controllable micro- and macro-structural features for cartilage TE purposes. Thermoplastic molds built by an inkjet printer were used to negatively replicate the design and form the TE scaffold, as described in our previous study (Figure 1) [22]. The struts of the negative mold were presented as channels in the final SF scaffold. SEM images also demonstrated that macro-scaled interconnected channels were successfully designed through indirect AM for cell migration and mass transport (Figure 1). Micro-pores were leaf-like in shape, and adjacent pores were generally in the same alignment. The channel width in the scaffold was approximately 740 μm, which could be compared with the diameter of the strut in the negative mold (700 μm). Morphologically, more condensed pores were noted along the channels, which could be attributed to the change in crystallinity of SF. As the scaffold constructs were exposed to boiling deionized water, the elevated ambient temperature and constant bombardment by the energetic surrounding water molecules may induce further β-sheet formation. This hence resulted in lesser porosity or densification of SF protein in the exposed regions [28]. The β-sheet crystalline conformation is converted from its random coil structure through ethanol immersion and thermal treatment. The presence of the crystalline secondary structure served to enhance the mechanical properties and insolubility in aqueous solution of the SF scaffold. It was speculated that the β-sheet content and topography of the SF constructs can induce better cell adhesion and spreading [7].

Micro-features, such as micro-pores within and surface properties of freeze-dried SF sponge in the scaffold, are expected to promote cell entrapment and attachment. The pores will serve as “pockets” to provide space for the cells to infiltrate, adhere and multiply. It was also observed that the micro-pores within the SF scaffolds exhibited similar morphologies to SF foams, further verifying that the incorporation of traditional freeze-dried manufacturing methods were compatible with indirect AM technology. Due to our combination of indirect AM technology with freeze-drying, the reproducibility of the SF scaffolds can be assured, as long as the process steps and conditions are preserved. It should be further emphasized that the reproducibility is combined with flexibility in user-defined control over CAD models, therefore allowing the customization of TE scaffolds to address patients’ needs in regards to shape and geometry.

**Figure 2.**
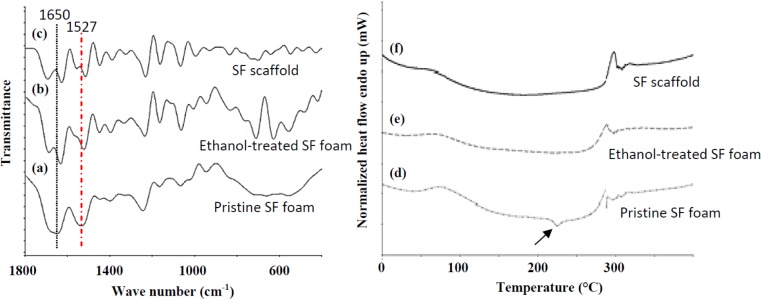
FTIR spectra (Lines **a**–**c**) and DSC (differential scanning calorimetry) thermograms (Lines **d**–**f**) of SF samples processed under different conditions. (**a**,**d**) Pristine SF foam; (**b**,**e**) ethanol-treated SF foam; (**c**,**f**) SF scaffold. The characteristic peaks of the SF scaffold were identical to ethanol-treated foam, but distinctive from those of pristine SF foam, as shown by the peak shifts of amide I and II (lines) in the FTIR spectra and the absence of an exothermic peak in the DSC thermograms (arrow).

The FTIR (Fourier transform infrared spectroscopy) spectrum of pristine SF foam (Figure 2, Line a) demonstrated characteristic amide I (1650 cm^−1^), II (1527 cm^−1^) and III (1241 cm^−1^) peaks that could be attributed to random coil protein conformation. Amide I and II peaks shift to 1617 and 1519 cm^−1^ after ethanol treatment in ethanol-treated SF foam (Figure 2, Line b) and the SF scaffold (Figure 2, Line c). Amide I and II peaks were recognized as a more obvious indication of β-sheet formation [29]. Thus, peak shifts confirmed the conformation structure change from the random coil to the β-sheet in ethanol-treated samples.

In the thermal analysis, the first endothermic peak between 50 and 100 °C was an indication of the breakage of hydrogen bonds between water molecules and SF polypeptide chains (Figure 2, Lines d–f). The SF scaffold and ethanol-treated SF foam were observed to have broader peaks than the pristine SF foam. The broadness of the peak was related to the amount of water present in each specimen [30]. Several pieces of literature have illustrated the process of heat energy release as an indicator of β-sheet formation when a protein sample is subjected to dynamic heating at a constant rate [30–32]. Thus, an exothermic peak at ~220 °C corresponding to this β-sheet formation during the heating process was observed for pristine SF foam (Figure 2, Line d). However, this peak was absent in the thermograms of ethanol-treated samples (SF scaffold and ethanol-treated SF foam) (Figure 2, Lines e and f), reconfirming β-sheet formation of SF after ethanol treatment. A second endothermic peak appeared in all samples by a sharp peak between 280 and 300 °C, which could be attributed to the thermal decomposition of the SF polypeptide chains. The endothermic peaks were also noted to be independent of the degree of the β-sheet structure within each sample. Moreover, there were no endothermic peaks seen at 50 and 95 °C, which represent the melting points of the support material and the thermoplastic mold, respectively. This observation suggests that the resultant SF scaffolds contain no or minimal traces of the sacrificial mold and its support material. Taken together, SF scaffolds fabricated by indirect AM technology preserve the intrinsic structure change feature induced by ethanol treatment of regenerated SF.

### 2.2. Cell Proliferation and Extracellular Matrix Production

**Figure 3.**
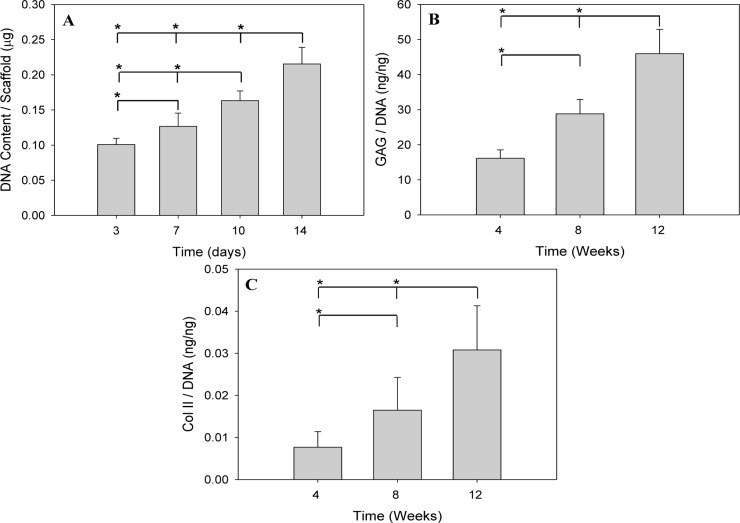
(**A**) Total DNA contents per scaffold; and (**B**,**C**) extracellular matrix (GAG and Col II) contents normalized to the DNA of the SF scaffold-chondrocytes constructs cultured *in vitro. N* = 6. * *p* < 0.05. GAG, glycosaminoglycan; Col II, collagen Type II.

Porcine chondrocytes were used in this study to assess the SF scaffolds’ potential for cartilage regeneration. Cells were first examined for their proliferation ability by measuring the DNA content in SF scaffolds. During the culture period, the cell number demonstrated a steady increase up to Day 14, as demonstrated by the significant increase of DNA content (*p* < 0.05) (Figure 3A). This observation is compatible with that of the traditionally manufactured, salt-leached 3D SF scaffold, where a cell proliferation up to Day 14 was noted [33]. For extracellular matrix (ECM) production, the amount of secreted glycosaminoglycan (GAG) and Col II per cell basis also showed a significant increase with time in Figure 3B,C. Considering the scaffolding material used in this study, the naturally-derived SF protein can emit appropriate biological signals for cell attachment and specific cell-biomaterial interactions and maintain the phenotypic expression of chondrocytes. In addition, the microstructural characteristics of the SF scaffold manufactured by indirect AM are recognized as important elements for the relevant cell signaling to regulate cell attachment, proliferation and differentiation [34].

Cartilage is composed of sparsely distributed chondrocytes embedded within a dense ECM, which is composed of primarily collagen Type II (Col II) and proteoglycans that provide the tissue with sufficient mechanical properties for function *in vivo*. Proteoglycans are composed of protein cores that are associated with one or more varieties of glycosaminoglycan (GAG) chains. Col II accounts for 90%–95% of the collagen in the matrix. Therefore, the differentiated phenotype of articular chondrocytes consists primarily of Col II and cartilage-specific proteoglycan. During serial monolayer culture, this phenotype is lost and replaced by a complex collagen phenotype consisting predominately of collagen Type I and a low level of proteoglycan synthesis. Such de-differentiated chondrocytes can re-express the differentiated phenotype in a suitable 3D environment with concomitant increase of proteoglycan and collagen synthesis [35]. That both GAG and Col II secretion were upregulated in Figure 3B,C suggest that the micro- and macro-morphological features of the SF scaffolds can promote the re-differentiation of chondrocytes, a crucial factor in cellular and tissue functional restoration [36].

### 2.3. Scanning Electron Microscopy and Confocal Microscopy Analysis

**Figure 4.**
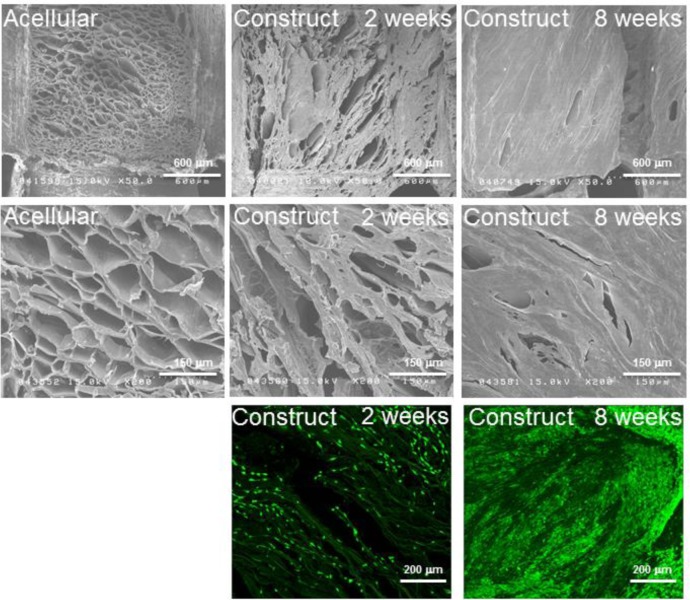
Scanning electron microscopic observations of the acellular SF scaffold (**A**,**D**), and SF scaffold-chondrocytes constructs (**B**,**C**,**E** and **F**) cultured *in vitro*. (**A**–**C**) top view (bar = 600 μm); (**D**–**F**) cross-section view (bar = 150 μm). The penetrating channels and surface channels could be seen from the top view; (**G**,**H**) Confocal microscopic observations of the SF scaffold-chondrocyte constructs cultured *in vitro* via the live/dead assays (bar = 200 μm). The leaf-like micro-structures of SF scaffolds are seen as the wavy lines dispersed between the round and bright green cells.

Neo-cartilage formation was examined by scanning electron microscopy (SEM) and confocal microscope in Figure 4. Cross-section views were taken to confirm the penetration of cells and successful ECM production at deeper levels of the scaffold. For the acellular scaffold, macro-channels were distributed at equal distances, and micro-pores exhibited leaf-like morphological details (Figure 4). For the SF scaffold-chondrocytes construct, ECM secreted by chondrocytes began to fill up the micro-pores within the scaffold, and individual cells were difficult to detect, as the ECM had a tendency to submerge the cells after two weeks of culture (Figure 4). By eight weeks of culture, the majority of the leaf-like micro-pores within the porous SF sponge were filled with ECM with the formation of a protruding and homogeneous slab of ECM within the inter-channel regions; however, macro-channels within the scaffold were preserved (Figure 4). The dense ECM layer was inferred to impede the cells’ migration into the channel area, hence attributing to the decreased amount of ECM secretion in this region. This observation was supported by a previous finding that suggested that the structural characteristics of the construct can influence cell proliferation and migration [37]. Moreover, in our previous study, optical coherence tomography imaging demonstrated cell attachment in designed channel areas, with a different spatial distribution and signal intensity compared to inter-channel regions, demonstrating varying cell behavior in different regions of indirect-AM designed scaffolds [20]. Nevertheless, it should be noted that the design of the macro-channels within the SF scaffolds serves to provide a pathway for the transport of essential nutrients and metabolic waste to the cells located in the central regions of the scaffold constructs. Moreover, these regions may be considered for further augmentation with vascular induction in the future. For native, non-vascularized tissues, such as cartilage, the pre-designed channels in the TE scaffold may serve as a viable solution for the lack of vascularized elements. For large tissue blocks, the preservation of these pre-designed channels indicates the ability to sustain nutrient and waste delivery. This aspect is foreseen to be increasingly crucial as the size of TE constructs increases [16,38].

The results obtained from confocal microscopy via the live/dead assays were consistent with the SEM images. Almost all chondrocytes showed green fluorescence, indicating high cell viability. After being cultured for two weeks, the distribution of the cells within the micro-pores exhibiting leaf-like morphology was even (Figure 4). After being cultured for eight weeks, the construct showed a vast increase of the cell number with micro-pores filled by cell aggregates (Figure 4).

**Figure 5.**
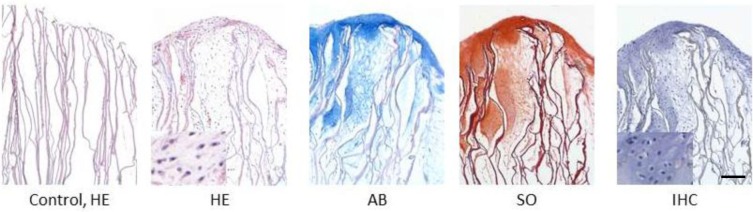
Histological analysis of the SF scaffold-chondrocytes constructs after eight weeks of *in vitro* culture (bar = 150 μm). HE, hematoxylin and eosin; AB, Alcian blue; SO, Safranin O; IHC, immunohistochemical stain of collagen Type II. The control is the acellular SF scaffold.

The morphology of chondrocytes, the distribution of cells and ECM synthesis in the SF scaffolds were further studied by hematoxylin and eosin (HE), Alcian blue, Safranin O and immunohistochemical (IHC) stains of the histological sections after eight weeks of *in vitro* cell culture (Figure 5). HE and IHC stains demonstrated oval or round-shaped cells in the SF scaffolds and showed the phenotypic characteristics of native chondrocytes (Figure 5, inserts). Cells had also penetrated into the leaf-like pores up to 900 μm and successfully secreted ECM within these pores. Positive Alcian blue and Safranin O stainings were seen on the histological sections with more intense staining in the top 500-μm region, indicating that cells were surrounded by sulfated proteoglycan-rich ECM, typical of cartilage. Consistent with the results in Figure 3C, Col II could be detected by IHC staining within the construct, demonstrating that chondrocytes seeded in SF scaffolds maintained their ability to synthesize and secrete cartilage ECM markers after long-term culture *in vitro*.

### 2.4. In Vivo Implantation

**Figure 6.**
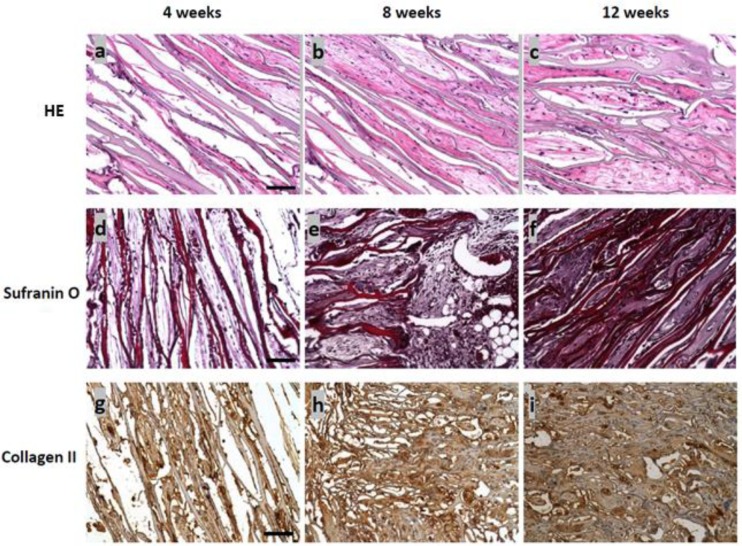
Histological analysis of the SF scaffold-chondrocyte constructs after 4, 8 and 12 weeks of subcutaneous implantation in nude mice (bar = 200 μm). Row 1: Hematoxylin and eosin (HE) stain; Row 2: Safranin O stain; Row 3: immunohistochemical stain of collagen II.

A subcutaneous implantation protocol was used to assess the ability of the seeded scaffolds to produce ectopic cartilage in an *in vivo* environment. The morphology of the chondrocytes, the distribution of cells and ECM synthesis were further studied by HE and Safranin O stains. Chondrocytes appeared round in shape, not spindle-shaped, the phenotypic characteristics of native chondrocytes, and were situated near the walls of the leaf-like pores. No apparent inflammatory cell infiltrates were noted, and adipose tissue infiltration of the scaffold was not seen, as well. However, capillaries had begun to invade by Week 4, suggesting an alternative source of nutrition and waste transport (Figure 6a). As the culture period reached eight weeks, a significant increase of the cell number was observed, and the distribution of chondrocytes within the micro-pores (and ECM) appeared to be homogeneous (Figure 6b). A distinguishable difference between the four-week and eight-week samples was the cell density within the interstitial matrix. More clustering of cells was noted, which may allow the cells to interact more and, thus, stimulate more ECM production [39]. The cell proliferation was further justified at 12 weeks, where the cell production was more extensive and cell clusters were seen between the micro-pores of the scaffold (Figure 6c).

The presence of GAG filled within the interstices of the ECM provided the swelling capabilities of the cartilage, hence enabling the connective tissue to resist compressive load. Histological staining by Safranin O revealed GAG production with respect to the implantation period (Figure 6d–f). The increase in staining intensity within the construct illustrates increased GAG detection, demonstrating the filling of cartilaginous tissue in the 3D SF constructs, as seen from SEM. Overall, the distribution of GAG was uniform at every time point.

Col II is one of the cartilaginous components synthesized by chondrocytes to build the ECM of connective tissue. The secretion of Col II also indirectly characterizes the chondrogenic phenotype of the regenerated connective tissue and plays an important role in the facilitation of chondrogenesis. Positive staining of this cartilage-specific marker was evident for the cell-seeded constructs for four, eight and 12 weeks. The immunochemical staining was found to be more widespread, as the constructs were implanted longer. In addition, it was noted that the tendency of Col II secretion was more prominent in wider pores up to eight weeks of culture (Figure 6g,h). The expression of the cartilage tissue marker was then observed to infiltrate into the smaller pores, which indicates the development of a stable infrastructure to support connective tissue growth (Figure 6i). Staining intensity was also higher around the scaffold material. This observation requires further investigation to better understand the cell-scaffold interactions. As a general observation from all time points, the distance between pore walls, which represents a marker for the onset of scaffold biodegradation, did not indicate any observable change. This suggests that degradation of the SF scaffold requires a longer time period than the observed 12 weeks under *in vivo* conditions.

## 3. Experimental Section

### 3.1. Preparation of Regenerated Silk Fibroin (SF)

Cocoons of *Bombyx mori* silkworm (Treenway Silks, Lakewood, CA, USA) were heated at 95 ± 5 °C for 30 min in an aqueous solution of 0.02 M Na_2_CO_3_ (Sigma-Aldrich, St. Louis, MI, USA). This process was repeated three times. The cocoons were then thoroughly rinsed with deionized water to ensure complete removal of the glue-like sericin protein. The degummed silk was left to dry for 24 h in a fume hood. SF solution was obtained by the dissolution of the degummed silk cocoons in a solvent mixture of CaCl_2_, ethanol and deionized water (molar ratio = 1:2:8) at 80 ± 5 °C for 4 h. This solution was then dialyzed in deionized water using a cellulose dialysis membrane (molecular weight cut-off = 6000–8000 Da) for 3 days. The deionized water was changed twice a day to ensure the complete removal of CaCl_2_. The diluted SF solution was subsequently centrifuged at 3500 rpm for 30 min and filtered to remove unwanted solutes. Finally, the SF solution was concentrated by dialyzing against 20% polyethylene glycol (molecular weight = 20,000 Da), and the concentration of the SF solution was determined by weighing the remaining SF solid after drying. The aqueous SF was stored at 4 °C until use.

### 3.2. Sacrificial Mold Fabrication

The negative sacrificial molds were designed using commercial CAD software (ProEngineer PTC, Needham, MA, USA) and manufactured using a Solidscape T612 Benchtop 3D inkjet printer (Solidscape Inc., Merrimack, NH, USA). The overall diameter and channel width of each negative mold was 10 mm and 700 μm, respectively. Using the droplet-based approach, each thin layer formed consists of two distinct materials, InduraCast™ (Solidscape Inc.) (thermoplastic) and InduraFill™ (Solidscape Inc.) (wax), to produce the mold and temporary supporting features, respectively. The support materials were removed by immersing the printed part in mineral oil at 60 °C, with constant stirring. The unwanted solutes and oil residues within the mold structures were blown off using an air gun. Subsequently, the molds were left to dry in a fume hood for 3 days.

### 3.3. Fabrication of SF Scaffolds and SF Foams

Aqueous SF with a concentration of 10 wt% was cast into the sacrificial thermoplastic mold. The concentration of SF solution was chosen based on the results from our previous study [22]. The cast molds were allowed to stand for 15 min under room temperature to ensure full penetration of the regenerated SF solution into the mold and were subsequently frozen at −80 °C for at least 12 h and freeze dried at −85 °C for 24 h to obtain porous SF sponges within the mold. After the lyophilization process, the specimens were immersed in ethanol (95% in methanol) for 20 min to induce the β-sheet structure transition of SF and insolubility in aqueous solutions. This procedure was followed by another 2 h of freeze drying before removing the molds. Upon achieving insolubility in aqueous solutions, the scaffolds were immersed in boiling deionized water to remove the thermoplastic mold materials, followed by freeze drying to yield scaffolds with defined macro-channels and morphological micro-features. To determine any possible changes of the physico-chemical properties of SF scaffolds during the indirect AM process, pristine and ethanol-treated SF foams were also prepared. The pristine foams were obtained after the sublimation of frozen 10 wt% SF solution. The ethanol-treated SF foams were obtained after subjecting these foams to the same ethanol and lyophilization treatments as the SF scaffolds.

### 3.4. Differential Scanning Calorimeter (DSC) and Fourier Transform Infrared (FTIR) Spectroscopy

The thermal properties of SF scaffolds and foams were analyzed using a Diamond DSC from Perkin Elmer (Waltham, MA, USA). The specimens of approximately 2.5 mg were sealed in an aluminum cell and heated at a heating rate of 10 °C/min from −30 to 400 °C under a nitrogen atmosphere. The chemical structure of SF were analyzed using an FT-730 FTIR spectrometer from Horiba (Kyoto, Japan). One milligram of each SF sample was pressed into a pellet with KBr, and the spectrum was recorded in transmittance mode with an accumulation of 10 scans with a resolution of 4 cm^−1^ and a spectral range of 4000 to 400 cm^−1^. The FTIR spectra obtained was de-convoluted using proprietary software to increase the peak separation.

### 3.5. Isolation and Culture of Porcine Chondrocytes

Porcine articular chondrocytes were isolated from articular cartilage derived from the knees of 4-week-old piglets. The experiments were approved by the Institutional Animal Care and Use Committee of Chang Gung University and adhered to the experimental care guidelines. The cartilage was dissected from the subchondral bone, rinsed with phosphate buffer solution (PBS) and minced into 1~2 mm^3^ pieces. The minced cartilage was digested by 0.2% collagenase in DMEM/Nutrient Mixture F-12 Ham (DMEM/F-12) (Sigma-Aldrich) supplemented with 10% fetal bovine serum (FBS, Thermo Scientific, Waltham, MA, USA). The digestion process was conducted at 37 °C with shaking for 12 h. The chondrocytes were collected by filtering the digestion solution through a sterile 70 μm nylon mesh (BD Falcon) to remove undigested fragments. Subsequently, the chondrocytes were washed with PBS twice and cultured at a density of 1 × 10^6^ cells/mL using DMEM/F-12 supplemented with 10% FBS, 1.12 g/L sodium bicarbonate and 50 mg/L ascorbic acid. The cells at Passage 2 were used for all experiments.

### 3.6. In Vitro Cell Culture in SF Scaffolds

SF scaffolds (10 mm in diameter and 5 mm in height) were sterilized with 70% ethanol and washed with PBS. The scaffolds were then immersed in cultural medium overnight before cell seeding. Each SF scaffold was placed in a well of a 24-well culture plate. A suspension of chondrocytes (1 × 10^7^ cell/mL) was seeded onto each SF scaffold, and cells were allowed to attach for 4 h at 37 °C. The cell-seeded scaffold was transferred to a new well with the addition of 1.5 mL of culture medium (DMEM/F-12 supplemented with 10% FBS) into each well. The cell culture was carried out at 37 °C in a humidified 5% CO_2_ incubator with a medium change every 3 days.

### 3.7. Biochemical Analysis

For the quantitative measurement of cell proliferation, SF specimens were harvested and digested at 60 °C for 24 h with papain solution (50 μg/mL with 55 mM sodium citrate, 150 mM sodium chloride, 5 nM cysteine HCl and 5 mM EDTA (ethylenediaminetetraacetic acid)). Total DNA content was measured by a DNA Quantitation Kit (Sigma-Aldrich) with calf thymus DNA as the standard. The digested solution was also used to quantify the amount of sulfated GAG present in the specimen. The amount of GAG was quantified using 1,9-dimethylmethylene blue reagent. Shark chondroitin sulfate was used as a standard to detect the GAG content, and measurements were recorded at 525 nm by a spectrophotometer. For the quantification of collagen Type II (Col II) content, the specimens were digested in 1% (w/v) pepsin in 0.05 M acetic acid and further solubilized with 0.1% (w/v) pancreatic elastase solution in Tris buffer saline (pH 8.0) at 4 °C for 24 h. The concentration of Col II was determined with a Type II collagen ELISA kit (MD Bioproducts) at 490 nm.

### 3.8. Scanning Electron Microscopy (SEM) Analysis

Scaffold-cell constructs were pre-fixed for 2 h in 3% glutaraldehyde, and the concentration of the fixative was changed to 5% for overnight fixation. The preserved SF constructs were rinsed with PBS and immersed in 1% osmium tetroxide in 0.1 M sodium cacodylate for 1 h at 4 °C. Following three rinses with PBS, the constructs were subjected to a series of ethanol treatments and air-dried. After drying, the specimens were placed on an aluminum holder, sputtered with gold and mounted on a Hitachi S-5000 SEM (Hitachi High-Technologies Co., Tokyo, Japan) for observation.

### 3.9. Confocal Microscopy Analysis

The Live/Dead Viability/Cytotoxicity Kit (Invitrogen, Carlsbad, CA, USA) was used to observe the viability of chondrocytes within the SF constructs. Specimens were washed with sterile PBS and immersed in PBS solution containing 4 mM calcein AM and 2 mM ethidium homodimer and incubated for 15 min. Subsequently, the constructs were observed under a Zeiss LSM 510 confocal microscope (Carl Zeiss AG, Jena, Germany).

### 3.10. In Vivo Implantation

For *in vivo* studies, subcutaneous implantation in nude mice was used to evaluate the biocompatibility of SF constructs and their potential in ectopic cartilaginous tissue formation. SF scaffolds were seeded with chondrocytes and cultured *in vitro* for 2 weeks, as described above, and the cell-scaffold constructs were implanted within the dorsal subcutaneous areas of six-week-old male athymic nude mice (SLC, Shizuoka, Japan). Implants were harvested at 4, 8 and 12 weeks for histological and immunohistochemical analysis. The number of animals used for the *in vivo* implantation was six.

### 3.11. Histological and Immunohistochemical (IHC) Analysis

After harvesting and washing with PBS, the specimens were fixed with 10% formalin for 24 h, embedded in paraffin, sectioned (5-μm thickness) and mounted on microscope slides. The sections were stained with hematoxylin and eosin (HE). Sulfated GAG in the harvested constructs was detected by Alcian blue and Safranin O stainings. For IHC analysis, expression of Col II was detected by mouse collagen Type II monoclonal antibody (Abcam, Cambridge, UK) and rabbit HRP-conjugated anti-mouse secondary antibody (Thermo Scientific).

### 3.12. Statistical Analysis

All data were expressed as the mean ± standard deviation. The data were statistically assessed with one-way analysis of variance (ANOVA), and Tukey’s post hoc test was used to compare between pairs of groups when significant variation was found. A *p*-value less than 0.05 was considered statistically significant.

## 4. Conclusions

This study demonstrated the fabrication of an SF TE scaffold with 3D interconnected macro-features by indirect AM technology for the facilitation of cellular-based mass transports. The micro-pores within the constructs served as biological cues to promote cell attachment and maintain cell morphology and function by the interactions of chondrocytes and SF. The pre-defined macro-channels facilitate cell migration towards the central regions of the scaffold and the effective mass transportation of nutrients and metabolic waste. The fine structural analysis of the 3D SF constructs revealed that the intrinsic properties, such as the thermal characteristics and chemical structure of SF, were preserved. Finally, the SF scaffolds have been proven as suitable substrates for cell proliferation and the expression of cartilage-specific ECM.
